# Novel Role of Dehydrated Human Amnion/Chorion Membrane in Healing of Denuded Ureter in Robot-Assisted Excision of Endometriosis

**DOI:** 10.1155/2019/1920430

**Published:** 2019-01-15

**Authors:** Osamah Hasan, Grant S. Johnson, James J. Siegert, Francisco Garcini, Thai T. Nguyen

**Affiliations:** ^1^Midwestern University Chicago College of Osteopathic Medicine, 555 31st St, Downers Grove, IL 60515, USA; ^2^Franciscan Health, 20201 South Crawford Avenue, Olympia Fields, IL 60461, USA; ^3^Silver Cross Hospital, 1890 Silver Cross Boulevard, Suite 210, New Lenox, IL 60451, USA; ^4^Advanced Urology Associates, 1541 Riverboat Center Drive, Joliet, IL 60431, USA

## Abstract

Ureteral injury is an uncommon but potentially morbid complication following any open or endoscopic pelvic procedure. Gynecologic surgeries alone make up 50 percent of nonurologic ureteral injuries leading to prolonged hospital stays, secondary interventions, and potential loss of renal function. The use of AmnioFix ® a processed dehydrated, immunologically privileged cellular amniotic membrane allograft has been well established in urologic and gynecologic procedures. These allografts contain human extracellular matrix components, growth factors, and cytokines that mediate inflammation and facilitate would healing. We report the first application of AmnioFix on a denuded ureter during a case of robotic-assisted excision of endometriosis. We include a literature review and discussion on the management outcomes of iatrogenic injury to the ureters.

## 1. Case Description

**Figure 1:**
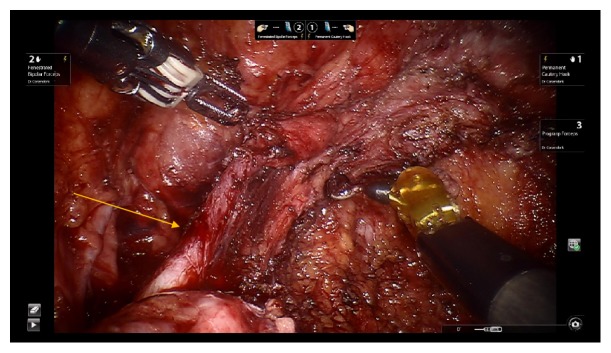
Intraoperative finding of left ureteral injury with subserosal hematoma (yellow arrow).

**Figure 2:**
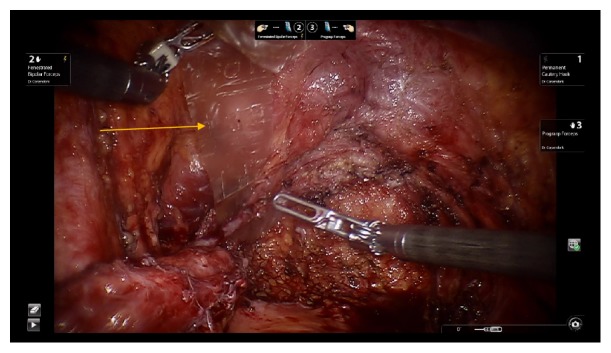
AmnioFix membrane wrapped around the site of left ureteral injury. AmnioFix membrane identified by yellow arrow.

**Figure 3:**
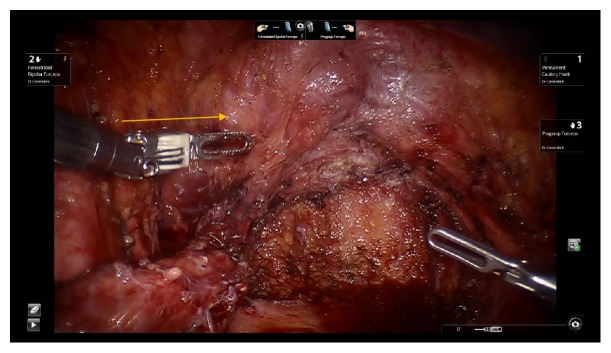
Left ureter following AmnioFix membrane placement. AmnioFix membrane identified by yellow arrow.

A 35-year-old Caucasian female with extensive history of pelvic surgery but without prior urological history underwent robotic-assisted laparoscopic excision of endometriosis by gynecological surgery team secondary to chronic pelvic pain with suspected endometriosis. On initial laparoscopic evaluation of pelvic contents, visible vermiculation of bilateral ureters was noted as well as suspected findings of endometriosis-like lesions covering the pelvic peritoneum. The pelvic peritoneum was excised with sparing of the urinary bladder. Careful ureterolysis was performed bilaterally, during which the distal left ureter was found to be partially denuded, spanning 2 cm in length (Figure 1). An intraoperative urologic consultation was requested, and denuded ureteral injury was confirmed by urology on laparoscopic evaluation. Given no evidence of ureteral laceration or obvious extravasation of urine from left ureter, no cystoscopy or contrast studies were performed. A 2 cm x 12 cm AmnioFix membrane was wrapped three times around the left ureter using laparoscopic robotic assistance (Figures 2 and 3). The procedure was completed without anesthesia complications and the patient was discharged on postoperative day one in stable condition.

**Figure 4:**
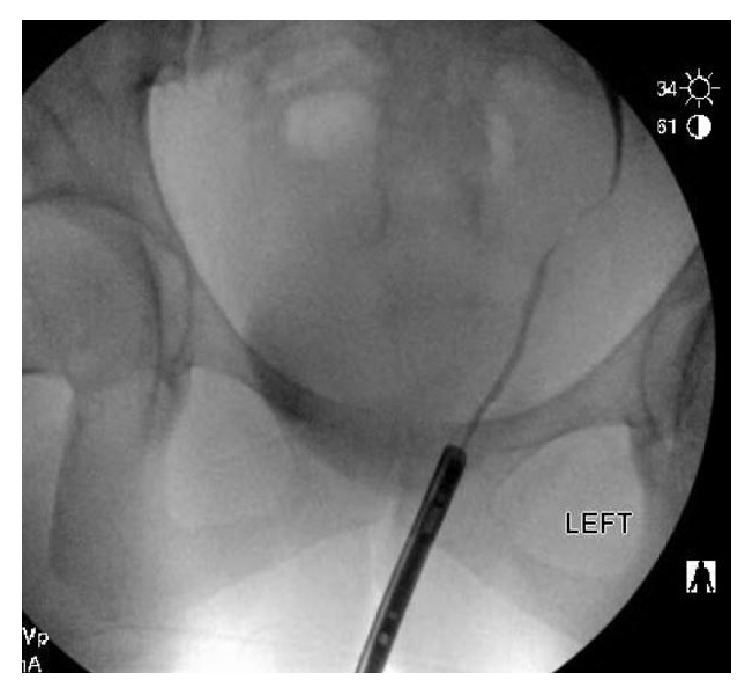
Left retrograde pyelogram shows 1.5 cm distal ureteral stricture with mild hydroureteronephrosis.

**Figure 5:**
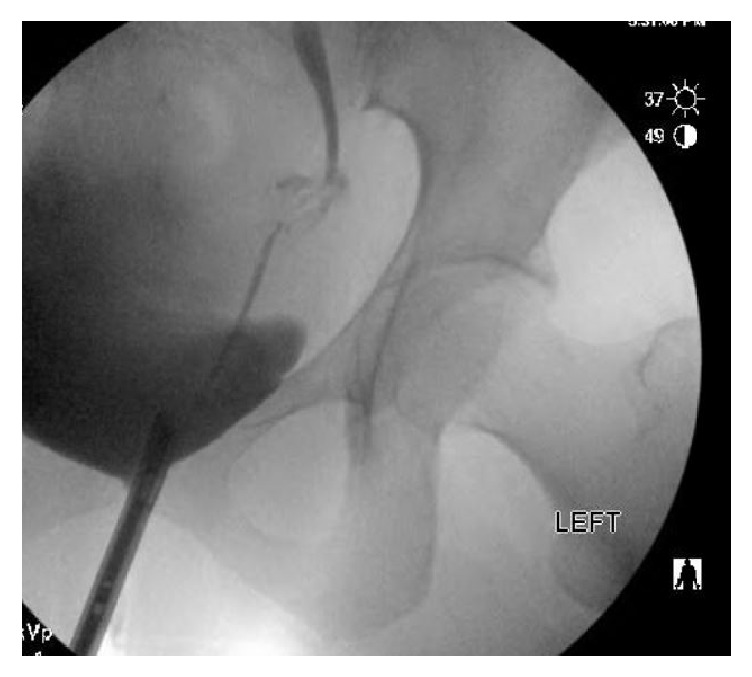
Mild extravasation noted on the left ureter with continued contrast injection.

The patient was seen by her gynecologist on postoperative day six after experiencing lower urinary tract symptoms and was subsequently started on PO antibiotic therapy. However, her symptoms did not improve, and she developed new left flank pain which brought her back to the hospital for further evaluation on postoperative day seven. She underwent noncontrast CT imaging of the abdomen and pelvis demonstrating moderate left hydroureteronephrosis to the level of the distal ureter. She underwent cystoscopy with left retrograde pyelogram demonstrating 1.5 cm distal ureteral stricture with moderate hydroureteronephrosis (Figure 4). Continued contrast injection showed a small amount of extravasation from the vicinity of the narrowed ureteral segment (Figure 5). However, the site of extravasation could not be delineated. A guidewire was passed through the left ureter and into left renal pelvis without resistance and a left ureteral stent was placed. Her pain improved, and she was discharged home.

Patient was readmitted one month later secondary to nausea, vomiting, and lower urinary tract symptoms at which time she was found to have enterococcus urinary tract infection. Cross section imaging of the abdomen and pelvis was unremarkable without fluid collections. Left ureteral stent was noted to be in appropriate position. She was discharged home with antibiotic therapy with outpatient follow-up in two weeks at which time her ureteral stent was removed.

**Figure 6:**
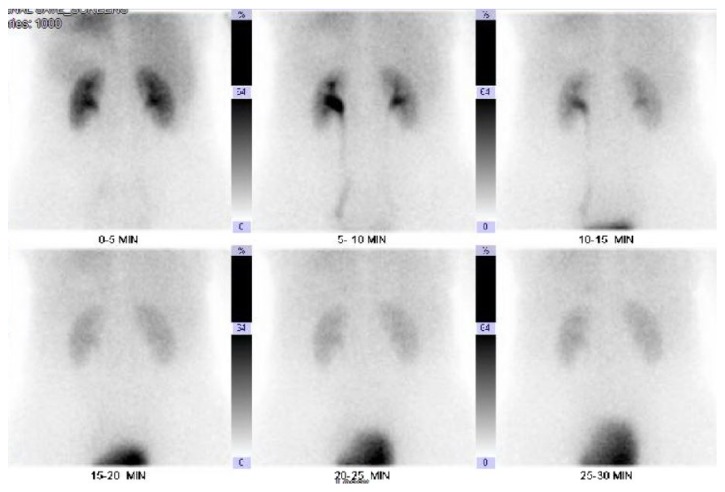
Renal scan: initial findings show mild asymmetrical activity in the left ureter compared to the right. However, washout at 20 min is symmetrical bilaterally without any signs of obstruction.

**Figure 7:**
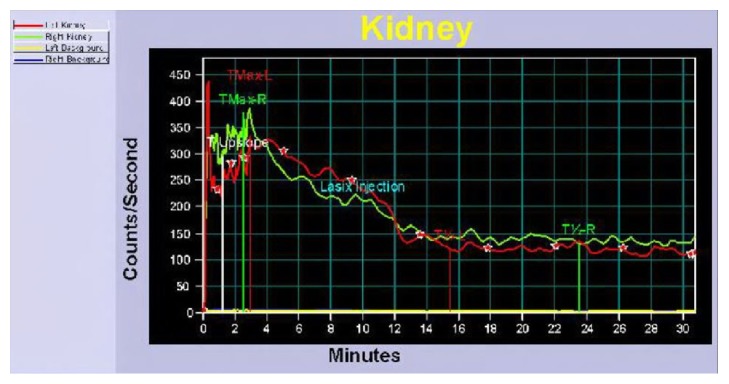
Renogram findings identical for left (red) and right (green) kidney: symmetrical downward slope consistent with no signs of obstruction bilaterally.

The patient did not report renal colic or abdominal pain following ureteral stent removal. A Lasix renal scan was performed three months following ureteral injury which demonstrated normal perfusion and excretion by 20 minutes without signs of left ureteral obstruction (Figures 6 and 7). Differential renal function was 45% left kidney and 55% right kidney. Repeat CT urogram performed 4 months after injury demonstrated no obstructive uropathy or contrast extravasation. Patient was recommended repeat Lasix renal scan in 1 year. The patient reported no symptoms during the interim.

## 2. Discussion

### 2.1. Incidence, Etiology, and Management of Ureteral Injury

While iatrogenic ureteral injuries are an uncommon complication of any abdominal or pelvic surgery, the incidence ranges from 0.2% to 1% [1]. Gynecologic procedures alone make up 50% of the ureteral injuries [1]. Lower anterior resection of the colon and abdominal perineal resection are responsible for 9% of ureteral injuries [2]. Assessment of an accurate incidence rate is likely hampered by unrecognized or unreported ureteric injuries [3]. Injury often occurs secondary to the close proximity of the ureter to the peritoneum and can occur in both complex and straightforward intra-abdominal and retroperitoneal surgery. Certain factors increase the risk of ureteral injury: endometriosis/endometriolysis, adhesions/adhesiolysis, distorted pelvic anatomy, coexistent bladder injury, lymphadenectomy, and vesicourethral anastomosis [3, 4].

The ureter is susceptible to injury along its entire course. However, the distal third of the ureter, from the pelvic brim to the bladder, is most frequently injured. Studies demonstrate that the most common site of injury is lateral to the uterine vessels [5]. Other common sites include the base of the infundibulopelvic ligament, at the level of the uterosacral ligament, and at the uterovesical junction [1]. Ureteric trauma can be injured from various mechanisms such as ligation or kinking by suture, crushing by a clamp, partial or complete transection, diathermy, or ischemia from devascularization. Signs and symptoms of ureteral injury usually include flank pain, hematuria, fever, retroperitoneal urinoma, and postoperative anuria [1].

In cases of suspected ureteral injury, intravenous pyelogram or contrast CT scan can assess for ureteric integrity and drainage. Intraoperative methods include infusion of indigo carmine with IV furosemide to assess for urinary extravasation [6]. Partial ureteral lacerations (i.e., ureteric defect <2.5 cm) are commonly managed by endoscopic placement of the stent for 2-6 weeks [7]. While approximately 50% of patients with lacerations showed no signs of obstruction at follow-up after stenting, the other half ultimately required ureteroneocystostomy (reimplantation) [7]. In our patient, a cystoscopy with retrograde pyelogram was performed to identify the anatomic site of obstruction. She was noted to have urinary extravasation and subsequently underwent ureteral stent placement.

### 2.2. Uses of AmnioFix in Surgical Applications

AmnioFix is composed of dehydrated human amnion/chorion membrane (dHACM), which is an immunoprivileged allograft containing an array of cytokines and growth factors that regulate processes involved in inflammation and wound healing. The dHACM allograft comes in multiple configurations and sizes to allow for its heterogenous use in internal and external applications. Various applications of AmnioFix have been well-described in literature. Subach et al. applied dHACM in patients undergoing lumbar fusion with posterior instrumentation effectively preventing epidural fibrosis and facilitating dissection in revision spinal surgery [8]. Mrugala et al. reported accelerated would healing after application of dHACM in five case reports in patients with chronic, nonhealing ulcers [9]. Dulemba et al. observed no adhesion formation in 14 of 15 cases of women undergoing robotic laparoscopy [10]. Patel et al. observed accelerated return of continence and potency in patients following nerve-sparing robot-assisted radical prostatectomy after the application of dHACM on the prostatic neurovascular bundle [11]. Ortega et al. in a poster presentation from a retrospective multicenter study showed dHACM significantly reduced the number of anastomotic leaks following colon resection [12]. These studies applied the dHACM in a unique format. Similarly, we employed AmnioFix in a novel manner with the express purpose of accelerating ureteric healing after ureteral denudement. Initial follow-up indicates that our patient has not required repeat intervention following ureteral stent removal and had a normal renal Lasix scan. The clinical goal was to apply AmnioFix in the hopes that the inherent growth factors would assist in healing, decrease ureteral stricture formation, and therefore avoid additional future surgical intervention such as ureteral reimplantation with neocystotomy.

## 3. Conclusion

Surgeons should be able to identify ureteral injury as soon as possible to improve surgical outcomes. The possibility of ureteric injury should be immediately considered if patients do not make satisfactory postoperative recovery or present with concerning signs or symptoms. Radiographic inquiries are essential to form the basis of diagnosis. This case report proposes a novel role for AmnioFix in ureteric injury in addition to standardized intervention as indicated. Given our patient's delayed injury and need for ureteral stenting, there is a possibility that AmnioFix exacerbated the injury or prolonged healing. Long term follow-up is planned to assess the absence or presence of ureteral stricture formation and subsequent need for surgical intervention. To accurately determine the clinical efficacy and utility of AmnioFix for ureteric healing, a randomized controlled trial would be required. Factors that might limit the widespread use of AmnioFix are high cost and lack of randomized controlled studies. This case hopes to assist surgeons in management of iatrogenic ureteral injuries by providing an additional option that could provide expedited healing, decrease morbidity, and ideally avoid future surgical intervention.
